# Diesel Exhaust Particles Exposure Disrupts Cardiac Bioenergetics and Induces a Shift Towards Glycolytic Metabolism in Rats

**DOI:** 10.3390/biom16101501

**Published:** 2026-10-15

**Authors:** Freddy G. Ganse, Marta Consegal, Cristina Rodríguez, Elisabet Miró-Casas, Marisol Ruiz-Meana, Javier Inserte, José Martínez-González, Ignacio Ferreira-González, Begoña Benito, Antonio Rodríguez-Sinovas

**Affiliations:** 1 Cardiovascular Diseases Research Group, Vall d’Hebron Institute of Research (VHIR) and Universitat Autònoma de Barcelona, Instituto de Investigación Sanitaria Hospital Universitario Vall d’Hebron (IIS IR-HUVH), 08035 Barcelona, Spain; freddy.ganse@vhir.org (F.G.G.); martaconse@gmail.com (M.C.); elisabet.miro@vhir.org (E.M.-C.); mruizmeana@gmail.com (M.R.-M.); javier.inserte@vhir.org (J.I.); iferregon@gmail.com (I.F.-G.); 2 CIBER de Enfermedades Cardiovasculares (CIBERCV), Instituto de Salud Carlos III (ISCIII), 28029 Madrid, Spain; crodriguezs@santpau.cat (C.R.); jmartinezg@santpau.cat (J.M.-G.); 3 Institut de Recerca Sant Pau (IR SANT PAU), 08041 Barcelona, Spain; 4 Instituto de Investigaciones Biomédicas de Barcelona-Consejo Superior de Investigaciones Científicas (IIBB-CSIC), 08036 Barcelona, Spain; 5 CIBER de Epidemiología y Salud Pública (CIBERESP), Instituto de Salud Carlos III (ISCIII), 28029 Madrid, Spain

**Keywords:** diesel exhaust particles, air pollution, myocardial metabolomics, metabolic reprogramming, mitochondrial dysfunction

## Abstract

Air pollution, particularly particulate matter (PM), is a major contributor to cardiovascular morbidity and mortality. We previously showed that diesel exhaust particles (DEPs) exposure increases arrhythmia susceptibility in rats, associated with myocardial oxidative stress and inflammation. However, the impact of DEPs on myocardial metabolism remains incompletely characterized. Here we aim to investigate whether DEP exposure induces time-dependent alterations in cardiac and plasma metabolic profiles following short- and longer-term intratracheal instillations in rats. Sprague–Dawley rats received intratracheal instillations of DEPs or saline three times weekly for one or three weeks. Myocardial and plasma metabolites were analyzed by ^1^H nuclear magnetic resonance spectroscopy, followed by discriminant and pathway analyses. Myocardial metabolomic profiling proved supportive of group separation, particularly one week after exposure. Seventeen of 22 metabolites were significantly altered. One week of DEP exposure reduced succinate, acetate, formate, 3-hydroxybutyrate, ATP, and ADP levels, a pattern consistent with alterations in mitochondrial oxidative metabolism and β-oxidation. Increased glucose and lactate levels were consistent with a greater reliance on glycolytic metabolism. Amino acid accumulation, along with increased inosine, pointed towards altered amino acid utilization and enhanced nucleotide degradation. Pathway analyses identified purine metabolism, glycolysis, citrate cycle, and pyruvate metabolism as some of the affected pathways, with enrichment of the citric acid cycle and mitochondrial electron transport chain. Most alterations resolved after three weeks. In contrast, changes in plasma were modest. In conclusion, DEP exposure induces transient metabolic remodeling in the myocardium, characterized by alterations consistent with reduced oxidative metabolism, increased glycolytic reliance, and disruption of energy and redox homeostasis, providing mechanistic insight into pollution-induced cardiac dysfunction.

## 1. Introduction

Air pollution is a major and modifiable determinant of cardiovascular morbidity and mortality, with particulate matter (PM) representing a key pollutant within urban environments [1]. Diesel exhaust particles (DEPs), rich in PM, form a carbonaceous aerosol enriched in redox-active organic compounds and transition metals capable of inducing oxidative stress and systemic vascular effects [2,3,4]. Multiple mechanisms link PM inhalation to cardiac injury, including pulmonary oxidative stress with systemic inflammation, direct translocation of smaller particles, and autonomic imbalance [3,4,5]. Consistently, genome-wide analysis of peritoneal macrophages from DEPs-exposed mice have demonstrated a systemic oxidative stress response [6], which ultimately translates into increased local oxidative stress and inflammation in the myocardium, as we have previously shown in rats exposed to DEPs for one or three weeks [2]. These alterations are associated with enhanced fibrosis and increased arrhythmia vulnerability, although not with overt cardiac dysfunction [2].

Oxidative stress and inflammation converge on metabolic pathways central to myocardial function. Therefore, characterization of the cardiac metabolome through metabolomics offers a powerful approach to identify biochemical mechanisms linking DEP exposure to cardiac dysfunction. Indeed, human studies provide evidence that diesel exhaust alters circulating metabolites relevant to cardiac physiology. In diesel engine testers, targeted serum metabolomics identified 22 metabolites associated with exposure, primarily within purine metabolism, including reductions in IMP, inosine, hypoxanthine and adenine [7]. Decreased serum cGMP further suggested disruption of the NO-cGMP signaling pathway, potentially contributing to endothelial dysfunction [7].

Traffic-related PM exposure has been associated with metabolic signatures indicative of mitochondrial and endothelial stress. In a cardiac catheterization cohort, short-term PM2.5 and ozone exposures induced delayed shifts in plasma metabolites, particularly within the glycine–ornithine–arginine axis and signatures of incomplete fatty-acid oxidation [8]. High-resolution plasma metabolomics have further identified alterations in amino acids such as arginine, alongside enrichment of pathways involved in mitochondrial bioenergetics (TCA cycle, glycolysis and pyruvate metabolism), oxidative stress responses, and nucleotide metabolism [9]. Similar pathway perturbations, including leukotriene and vitamin E metabolisms, have been reported in the DRIVE study (analysis performed in samples from plasma or saliva), together with consistent changes in metabolites as arginine, histidine, γ-linolenic acid, and hypoxanthine [10]. Controlled exposure studies comparing high- versus low-traffic environments also reported changes in circulating acylcarnitines, phosphatidylcholines, and amino acids as tyrosine [11].

Experimental models support these observations. In mice exposed to vehicle emissions, serum metabolomics revealed increased metabolites in the cysteine–glutathione synthesis pathway, elevated oxidized linoleate derivatives, and altered vasoactive mediators, including increased bradykinin and decreased adenosine [12]. Similarly, PM2.5-exposed rats showed significant metabolic changes in serum and urine, affecting pathways such as glycine, serine and threonine metabolism, glyoxylate and dicarboxylate metabolisms, the TCA cycle, and nitrogen and methane metabolisms [13]. These systemic metabolic alterations may provide a mechanistic link between inhaled DEPs and cardiovascular outcomes.

At the cellular level, emerging evidence indicates direct effects on cardiomyocyte bioenergetics. In HL-1 mouse cardiomyocytes exposed to diesel-derived PM2.5, integrated transcriptomics and metabolomics revealed disrupted amino-acid profiles, increased reactive oxygen species (ROS) production, impaired ATP synthesis, and disrupted Ca2+ homeostasis, accompanied by structural mitochondrial damage [14]. Together, these findings support a model in which DEP or PM exposure induces mitochondrial dysfunction and redox imbalance, ultimately impairing cardiac performance.

Despite these advances, key gaps remain, including limited characterization of the myocardial metabolome following controlled DEP exposure and insufficient temporal resolution to distinguish short- from long-term adaptations. Accordingly, the present study aims to determine whether DEP short-term and prolonged DEP exposure induce alterations in cardiac and plasma metabolic profiles, with particular emphasis on pathways related to energy metabolism, mitochondrial function, and oxidative stress.

## 2. Materials and Methods

All animal procedures were conducted in compliance with the European Directive 2010/63/EU for the protection of animals used in scientific research and the NIH Guide for the Care and Use of Laboratory Animals (NIH Publication No. 85-23; 1996 revision, updated 2011). The experimental protocol was approved by the Ethics Committee of the Vall d’Hebron Research Institute (protocol CEEA49.20, CEA/11228/P1/1).

The samples analyzed in the present work were obtained from animals included in a previous study [2]. Therefore, no additional animals were used, and the sample size was determined by the number of biological samples available from the original experiments. This approach also adhered to the principle of reduction by maximizing the scientific information obtained from previously collected samples.

### 2.1. Study Protocol

Six-week-old Sprague–Dawley rats of both sexes, with free access to food and water, were included in the study. Animals were allocated to the experimental groups maintaining an approximately equal distribution of males and females within each group, as previously described [2]. After a four-week acclimation period, animals were randomly assigned to receive intratracheal instillations of either saline or saline containing diesel exhaust particles (DEPs; 7.5 mg/Kg, 0.375 mL/kg). Procedures were conducted under general anesthesia induced by ketamine (75 mg/Kg, IP) and medetomidine (0.5 mg/Kg, IP), with anesthesia subsequently reversed using atipamezole (1 mg/Kg, IP). DEP treatments were administered three times weekly over periods of either one or three weeks [2]. A single saline-treated control group was included in the study and was exposed to the same experimental procedures for 3 weeks, corresponding to the longest exposure period used in DEP-treated animals. This design was intentionally chosen to control for any potential effects associated with repeated intratracheal instillations, anesthesia, handling, and the overall duration of the experimental protocol under the most prolonged conditions. At the end of the exposure period, plasma samples were obtained, animals were euthanized, and hearts were rapidly excised. Ventricular tissue was isolated, and together with the plasma samples, was snap-frozen in liquid nitrogen, and stored at −80 °C until further analysis. The DEP dose regimen was adapted from protocols described previously [15,16] and adjusted according to the body weight of the animals [2].

DEPs (DEPs; SRM-2975, National Institute of Standards and Technology, Gaithersburg, MD, USA) were prepared as a stock suspension (20 mg/mL) in sterile 0.9% saline. According to the manufacturer, mean particle diameter (area distribution) is 11.2 ± 0.1 μm, with a surface area of 0.538 ± 0.006 m^2^/cm^3^. Previous studies have detected that they contain high levels of elemental carbon, as well as variable amounts of inorganic ions and metals, but lack polycyclic aromatic hydrocarbons [17]. Further details on particle characteristics can be found at the National Institute of Standards and Technology web page (www.nist.gov). Prior to use, suspensions were vortexed and sonicated to minimize particle aggregation. The rationale for the choice of the DEP dose is described in detail in [2].

### 2.2. Analysis of Myocardial Metabolism

Plasma (100 μL) and cardiac (50 mg) metabolites were extracted using the methanol and methanol:chloroform methods, respectively [18]. The resulting extracts were reconstituted in 600 μL of deuterium oxide containing 1 mmol/L of TSP (3-(trimethylsilyl)propionic-2,2,3,3-d4 acid) (TSP; Sigma-Aldrich, St. Louis, MO, USA) as an internal standard. ^1^H NMR spectra were acquired using a 5 mm probe tuned for proton detection in a vertical bore 9.4T magnet coupled to a Bruker Avance 400 spectrometer (Bruker Corporation, Billerica, MA, USA) [18]. Spectra were collected using a Carr Purcell Meiboom Gill (CPMG) pulse-and-acquire sequence with water signal presaturation, accumulating 64 scans per sample, with a an effective T2 delay of 32 ms. Continuous wave irradiation was used to suppress water resonance. Sweep width was set to 11 ppm and an inter-pulse delay of 20 s was applied between scans. Spectra processing and peak fitting were performed using the XWINNMR software (version 3.5; Bruker Corporation, Billerica, MA, USA). Following multiplication by a 0.2 Hz exponential function, spectra were Fourier-transformed and manually phase-corrected. Metabolite identification was based on characteristic chemical shifts and signal multiplicities, and metabolite concentrations were determined by comparison with the signal area of the 1 mM TSP standard. Spectral quality was assessed prior to analysis by inspection of baseline stability, line shape, chemical shift alignment, and signal-to-noise ratio.

Spectra were integrated between 0.5 and 9 ppm, excluding the residual water region (4.5–5.5 ppm). Spectral data were normalized to the total spectral area and imported into the SIMCA-P software (Sartorius Stedim Data Analytics AB, Umea, Sweden). Prior to orthogonal partial least squares discriminant analysis (OPLS-DA), variables were subjected to pareto scaling to reduce the influence of variables with high noise levels, while preserving biological variation. For Pareto scaling each variable was scaled to (1/Sk)1/2, where s is the standard deviation of the variable (integral region) k.

### 2.3. Statistical Analyses

Data are presented as mean ± standard error (SEM). Statistical analysis was performed using GraphPad Prism 9.4.0. Normality was assessed using the Shapiro–Wilk test and homogeneity of variance using the Brown–Forsythe test. Differences in metabolite concentrations between control and DEP-exposed animals, either for one or three weeks, were initially assessed using a one-way ANOVA, except for those that did not satisfy the assumptions required for parametric testing (3-hydroxybutyrate, glutamate, inosine and succinate in myocardial samples). In these cases, analysis was performed using the Krustal–Wallis test. To account for multiple comparisons across metabolites, *p* values were adjusted using the Benjamini–Hochberg false discovery rate (FDR) procedure. Differences between groups were further analyzed by one-way analysis of variance (ANOVA) and Fisher LSD post hoc tests and OPLS-DA using SIMCA 14.0 (Sartorius Stedim Dana Analytics AB, Umea, Sweden). For Pathway Analysis and Enrichment Analysis, all 22 metabolites identified were entered into MetaboAnalyst 6.0. Pathway analyses were performed to identify metabolic pathways associated with the observed metabolomic profile. These analyses were intended to provide biological context for the metabolite changes and not to infer direct pathway activation or inhibition. Differences were considered significant when *p* < 0.05.

## 3. Results

### 3.1. Myocardial Metabolic Profile of Rats Exposed to DEPs for One or Three Weeks

**Figure 1:**
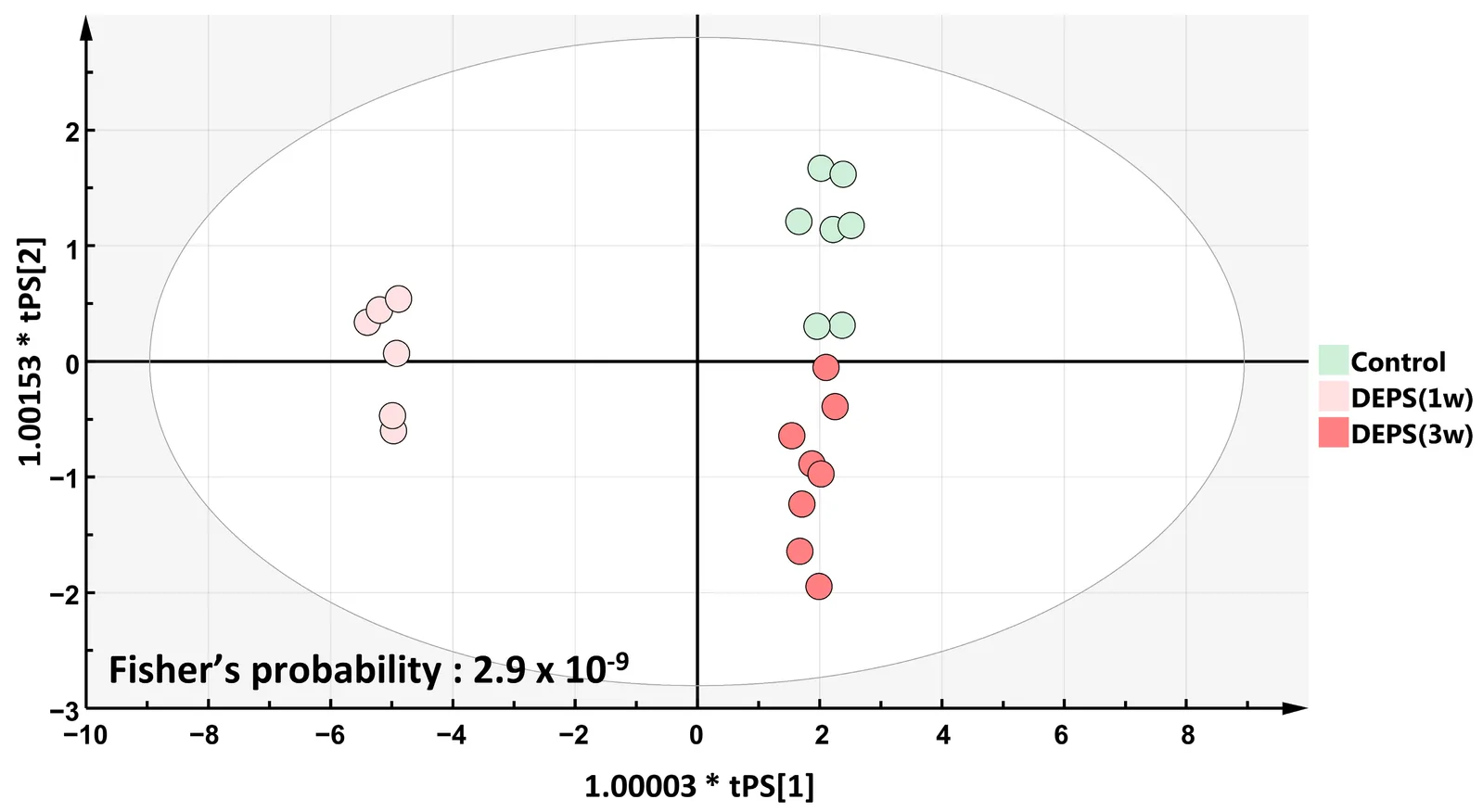
Score plot corresponding to the discriminant analysis (OPLS-DA) of metabolites determined in myocardial samples from control rats (n = 7) and from animals exposed to DEPs for one (n = 6) or three (n = 8) weeks.

Supervised classification of ^1^H NMR spectra acquired from myocardial extracts from rats intratracheally instilled with either saline or with DEPs for one or three weeks provided supportive evidence of discrimination among treatment groups (Fisher test: *p* = 2.9 × 10^−9^) (Figure 1). Model validation yielded R^2^ and Q^2^ values of 0.83168 and 0.71271, respectively, with a cross-validated classification accuracy of 0.66667. Permutation testing (100 permutations) confirmed that the observed separation was unlikely to occur by chance (empirical *p* < 0.01). This separation was particularly pronounced in animals exposed to DEPs for one week. These findings indicate that the different treatment groups may exhibit distinct cardiac metabolic profiles.

**Figure 2:**
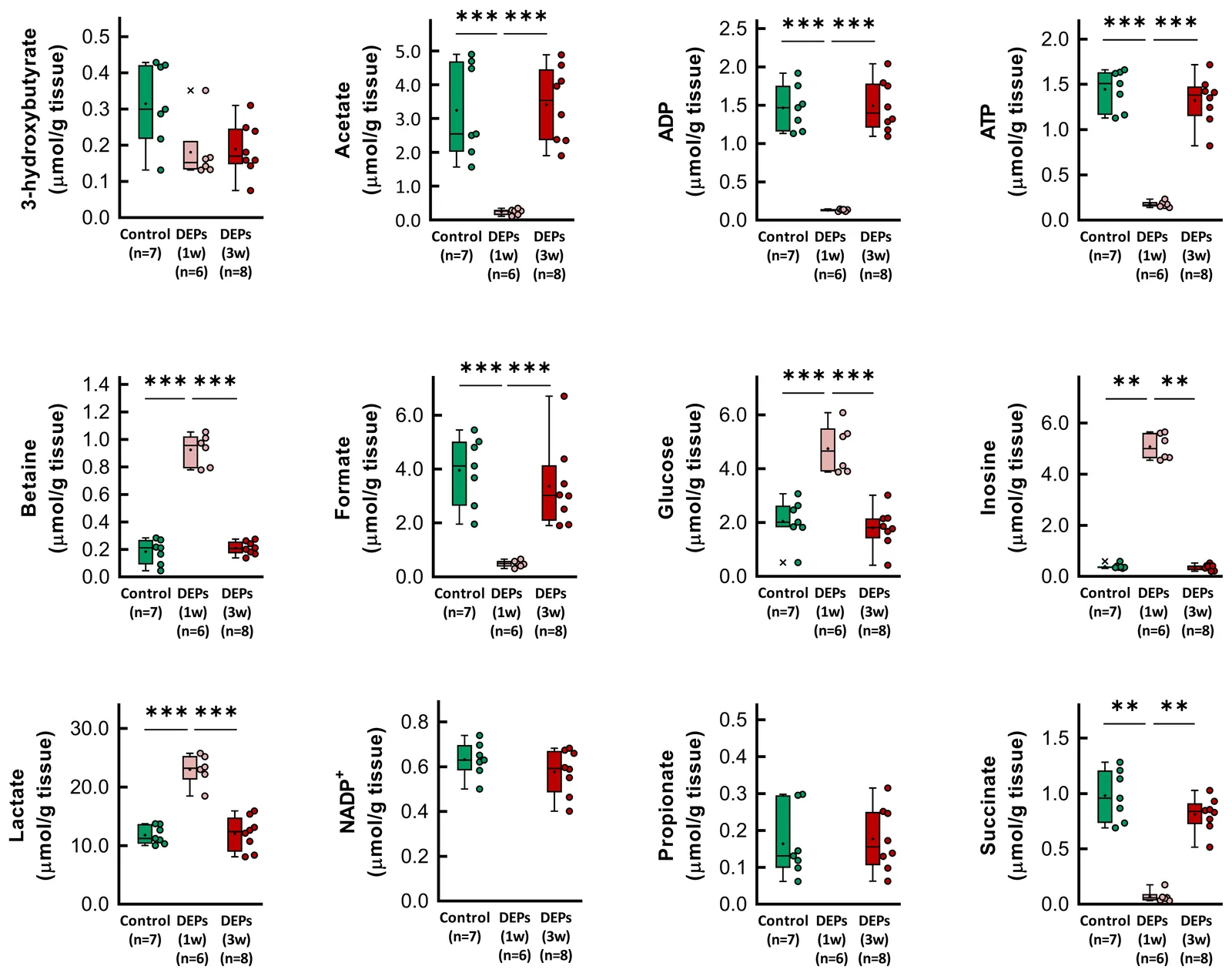
Quantification of metabolites by ^1^H NMR spectroscopy in myocardial samples from control rats (n = 7) and from animals exposed to DEPs for one (n = 6) or three (n = 8) weeks. Values for NADP^+^ and propionate at one week of exposure were below detection limits. ** (*p* < 0.01) and *** (*p* < 0.001) indicate significant differences between indicated groups after Benjamini–Hochberg FDR correction (ANOVA test). Data are shown as box plot depicting median (horizontal line), mean (+), individual values (color symbols) and outliers (x).

**Figure 3:**
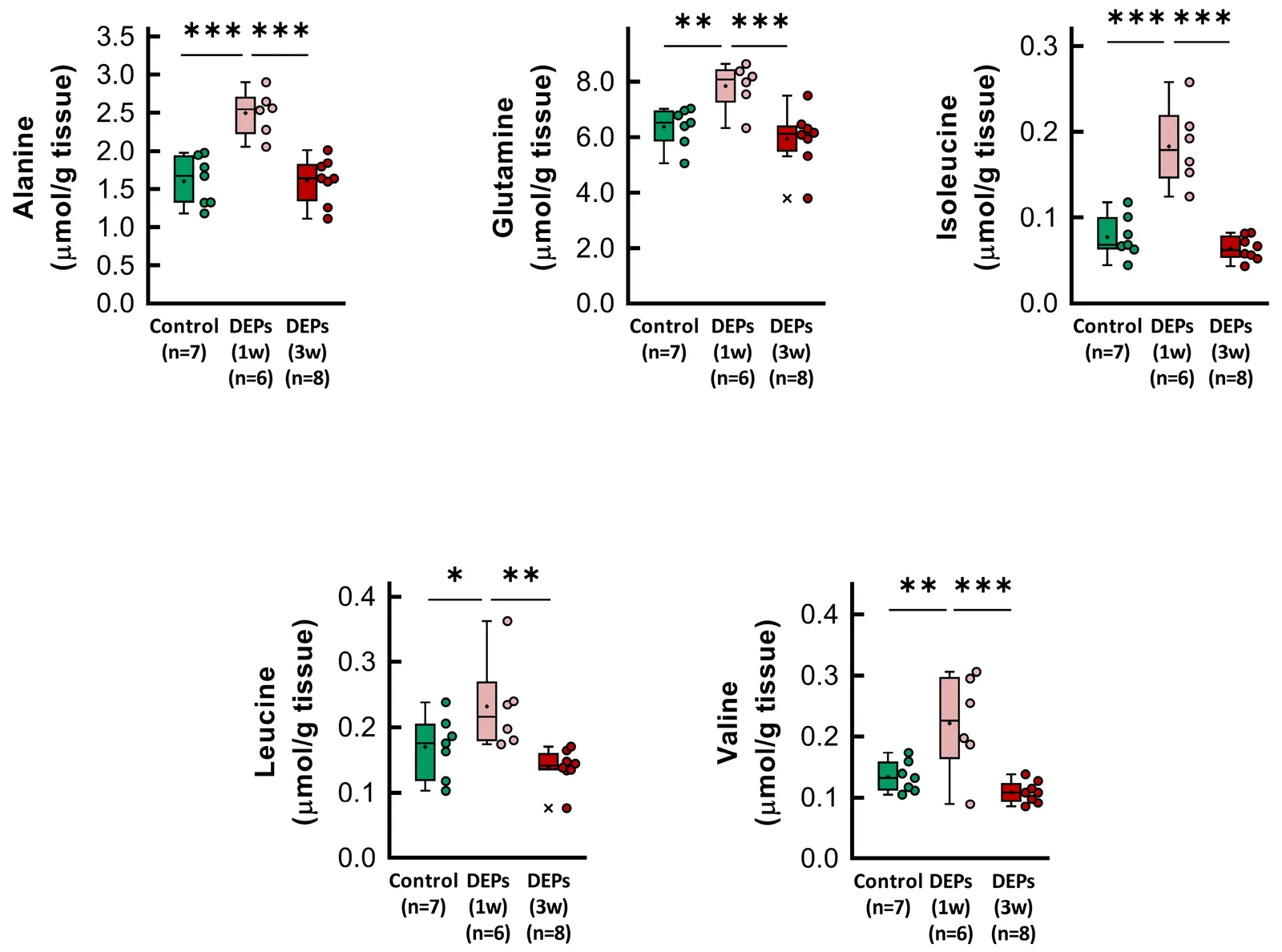
Quantification of selected amino acids by ^1^H NMR spectroscopy in myocardial samples from control rats (n = 7) and from animals exposed to DEPs for one (n = 6) or three (n = 8) weeks. * (*p* < 0.05), ** (*p* < 0.01) and *** (*p* < 0.001) indicate significant differences between indicated groups after Benjamini–Hochberg FDR correction (ANOVA test). Data are shown as box plot depicting median (horizontal line), mean (+), individual values (color symbols) and outliers (x).

Seventeen of the 22 quantified metabolites differed significantly across treatment groups (Figure 2, Table 1). In hearts from animals exposed to DEPs for one week, concentrations of acetate, ADP, ATP, formate and succinate were significantly reduced, with NADP^+^ and propionate being below detection limit in this group only. In contrast, concentrations of betaine, glucose, inosine, and lactate were markedly increased at this time point (Figure 2, Table 1). Similarly, the ATP/ADP ratio was increased at one week of exposure (Table 1). Regarding amino acids, alanine, glutamine, isoleucine and valine were significantly increased one week after exposure, with a similar trend for leucine (Figure 3, Table 1). Notably, most of these changes reverted towards baseline three weeks after DEP exposure (Figure 2 and Figure 3, Table 1). Leucine levels three weeks after exposure were significantly lower compared with those found in hearts from animals treated for one week (Figure 3, Table 1).

**Figure 4:**
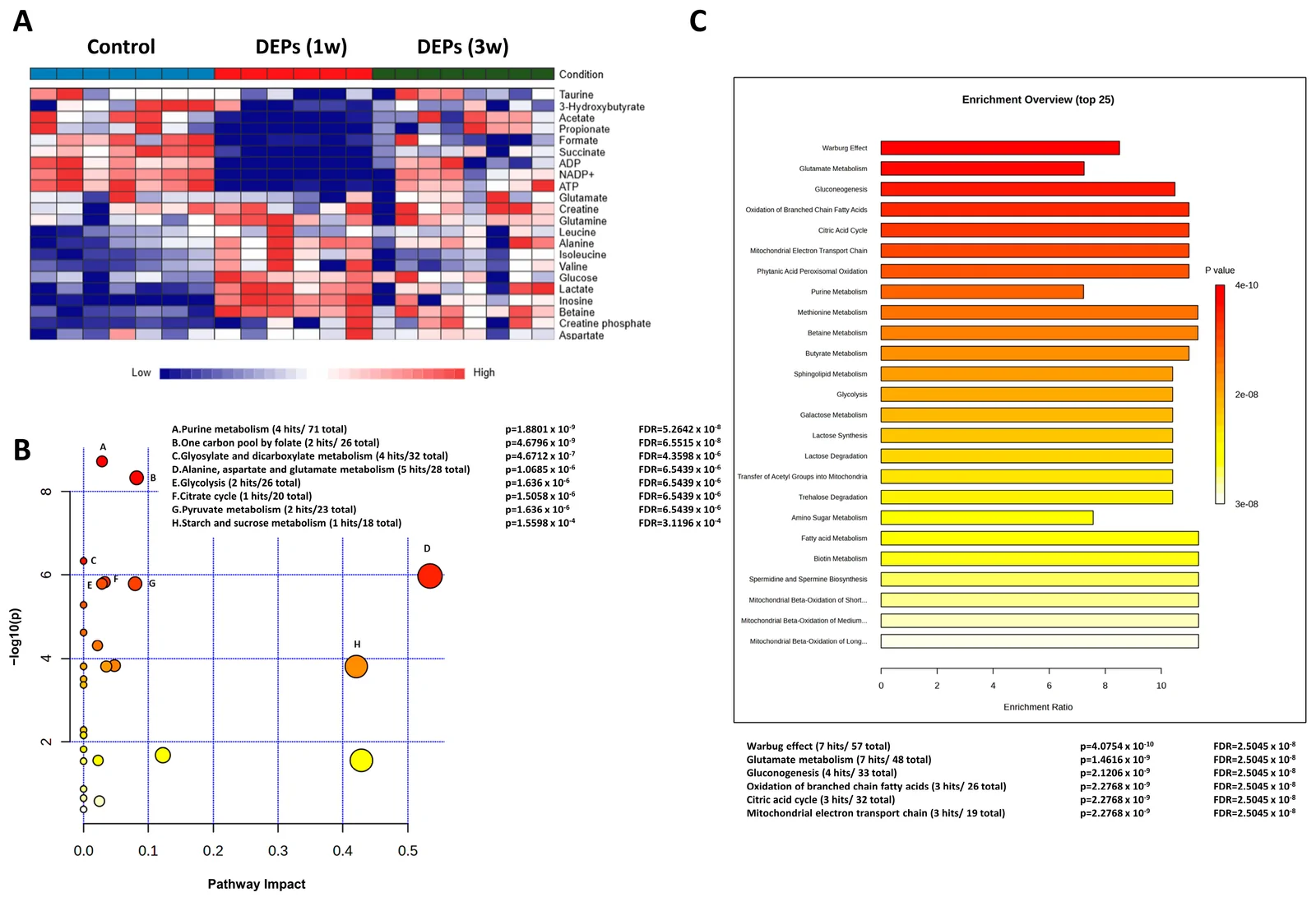
Non-targeted metabolomic analysis of myocardial samples from control rats (n = 7) and from animals exposed to DEPs (n = 6 and n = 8 for those exposed for one and three weeks, respectively). (**A**) Heat-map of metabolites between individual cases in myocardial samples from all three groups of animals. (**B**) Pathway analysis of identified metabolites for animals exposed for one week. (**C**) Pathway enrichment analysis of identified metabolites. FDR: False Discovery Rate.

The heat-map shown in Figure 4A shows changes in metabolites between individual cases in hearts from control animals and from rats instilled with DEPs for one or three weeks. Pathway analysis suggested multiple altered pathways in hearts from animals exposed to DEPs for one week, as compared with those from control animals, including purine metabolism, glyoxylate and dicarboxylate metabolism, alanine, aspartate and glutamate metabolism, glycolysis, citrate cycle, and pyruvate metabolism (Figure 4B). Pathway enrichment analysis suggested more than an eight-fold enrichment in Warburg effect, gluconeogenesis, oxidation of branched chain fatty acids, citric acid cycle, mitochondrial electron transport chain, and phytanic acid peroxisomal oxidation, among others (Figure 4C).

### 3.2. Plasma Metabolic Profile of Rats Exposed to DEPs for One or Three Weeks

**Figure 5:**
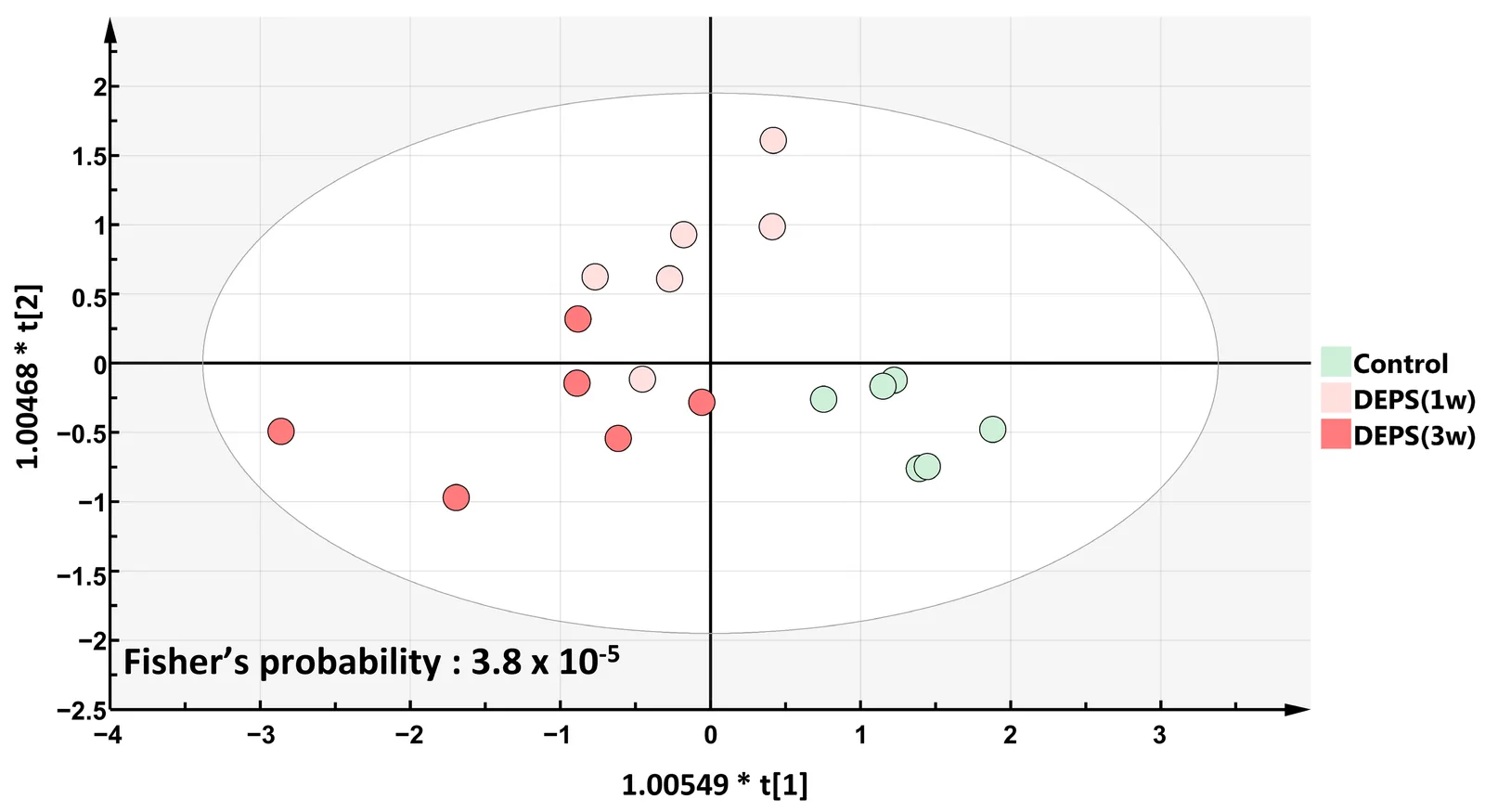
Score plot corresponding to the discriminant analysis (OPLS-DA) of metabolites determined in plasma samples from control rats and from animals exposed to DEPs for one or three weeks (n = 6/group).

**Figure 6:**
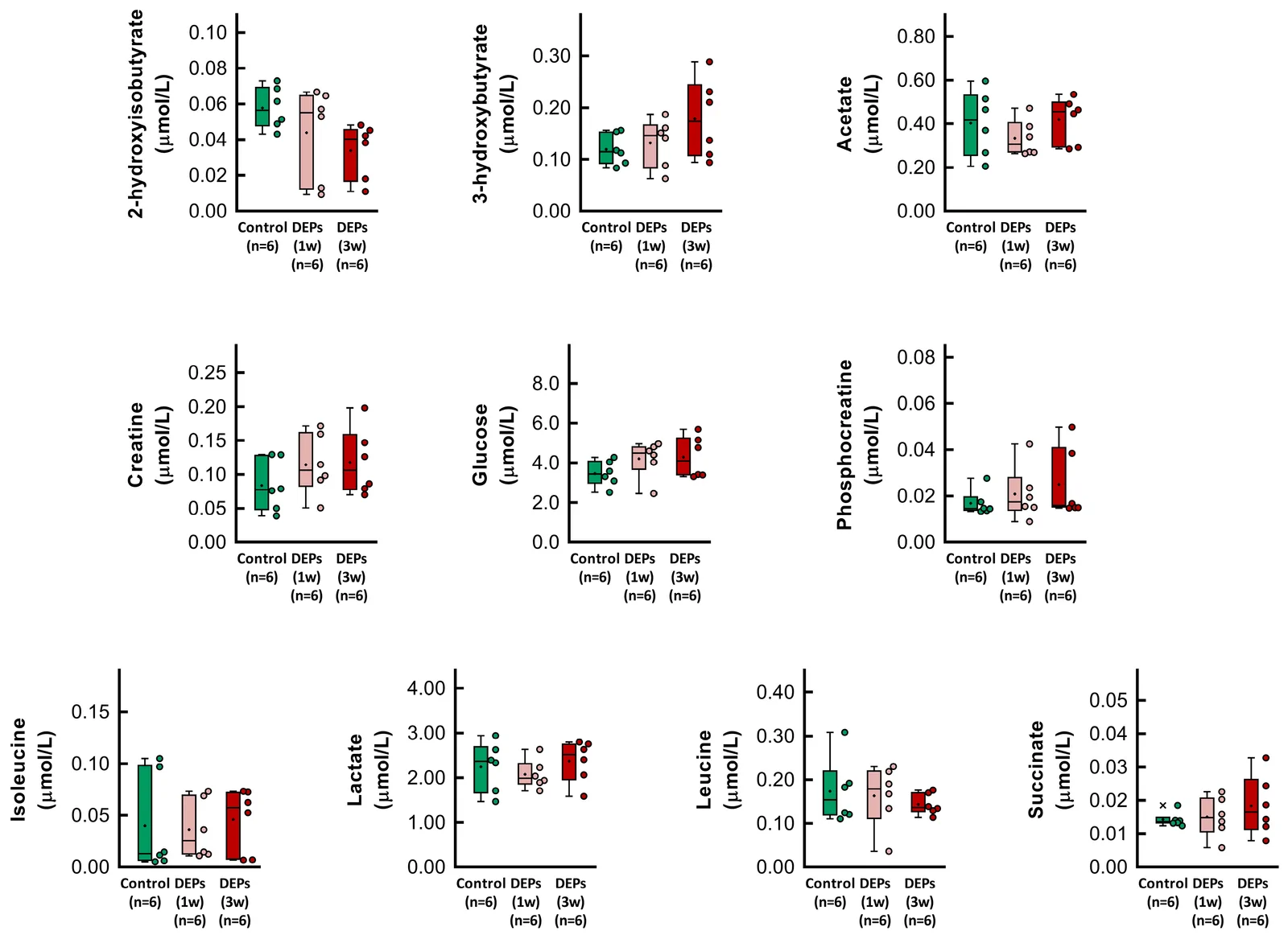
Quantification of metabolites by 1H NMR spectroscopy in plasma samples from control rats and from animals exposed to DEPs for one or three weeks (n = 6/group). No significant differences were observed in any case. Data are shown as box plot depicting median (horizontal line), mean (+), individual values (color symbols) and outliers (x).

Differences in the metabolic profile of plasma samples were less apparent than those found in myocardial extracts. Despite this, supervised classification of ^1^H NMR spectra was still able to provide supportive evidence of separation between groups (Fisher test: *p* = 3.8 × 10^−5^) although model validation yielded poor R^2^ and Q^2^ values (Figure 5). No significant differences were found in any of the 10 metabolites identified, although a trend towards a reduction in 2-hydroxyisobutyrate and an increase in 3-hydroxybutyrate was observed three weeks after DEP exposure (Figure 6).

## 4. Discussion

Our present results indicate that short-term exposure to DEPs induces acute alterations in the myocardial metabolic profile, which largely resolve over the long-term. Pathway analysis suggested purine metabolism, glyoxylate and dicarboxylate metabolism, alanine, aspartate and glutamate metabolism, glycolysis, citrate cycle, and pyruvate metabolism as the most affected pathways. In addition, pathway enrichment analysis suggested enrichment in Warburg effect, gluconeogenesis, oxidation of branched chain fatty acids, citric acid cycle, mitochondrial electron transport chain, and phytanic acid peroxisomal oxidation. Notably, marked decreases were observed in acetate, ADP, ATP, formate and succinate levels, whereas increases were detected in metabolites associated with purine metabolism, as inosine. In contrast, metabolic alterations in plasma were comparatively modest.

We have previously demonstrated that intratracheal instillation of DEPs in rats induces a pro-arrhythmic effect in isolated hearts three weeks after exposure [2]. This effect was associated with moderate interstitial collagen deposition, myocardial inflammation, sustained oxidative stress, and activation of specific cytosolic signaling pathways [2]. Most of these alterations appeared to represent early responses to exposure, as evidenced by changes in several mRNA markers, including those associated with fibrosis and inflammation [2]. The present findings suggest a similar pattern in myocardial metabolism, with pronounced alterations at one week that largely resolve by three weeks of exposure. Taken together, this pattern may indicate that DEP exposure induces early myocardial alterations, including metabolic disturbances, that are at least partially compensated over time, rather than a progressive worsening. Thus, the metabolic profile observed after 1 week may represent an early adaptation to oxidative and inflammatory stress, promoting ATP production through glycolysis when mitochondrial function is challenged. The attenuation of these changes after 3 weeks suggests the development of compensatory mechanisms that partially restore metabolic homeostasis despite continued exposure. These findings indicate that DEP-induced metabolic remodeling is dynamic and evolves over time.

An additional question raised by these findings concern the relationship between the apparent metabolic recovery observed after 3 weeks of DEP exposure and the mitochondrial alterations previously reported in this model [19]. One possibility is that the attenuation of the metabolomic changes reflects the activation of compensatory mechanisms that partially restore oxidative metabolism and metabolic homeostasis despite continued exposure. In this context, the previously observed reduction in citrate synthase activity, decrease in mitochondrial DNA copy number, and increase in mitochondrial ROS production [19] could be related to the energetic demands associated with this adaptive process. However, the present data do not allow the establishment of a temporal sequence or causal relationship between metabolic remodeling and mitochondrial alterations. Therefore, it remains unclear whether the mitochondrial changes drive the metabolic adaptations, whether the metabolic adaptations contribute to mitochondrial dysfunction, or whether both processes arise in parallel as part of the myocardial response to DEP exposure.

Most metabolomic studies on PM exposure have focused on the analysis of plasma or serum samples, which may reflect systemic effects. However, relatively few studies have examined direct myocardial metabolic responses to air pollutants. To our knowledge, only one study in mice exposed to concentrated ambient PM2.5 has reported alterations in nitric oxide signaling and muscle-contraction-associated pathways [20].

Previous proteomic studies suggested that particulate matter disrupts, in rat hearts, key cardiac processes, including the citric acid cycle, ATP synthesis, and oxidative phosphorylation [21]. Additional support for mitochondrial involvement comes from our recent work using the same experimental model, in which DEP exposure reduced citrate synthase activity in both subsarcolemmal and interfibrillar mitochondria, decreased mitochondrial DNA copy number, and increased mitochondrial ROS production [19]. These findings are consistent with reduced mitochondrial content and altered mitochondrial function, providing independent evidence that complements the metabolic alterations reported in the present study. Our metabolomic data are consistent with these observations and are suggestive of a shift from oxidative to glycolytic energy metabolism [22,23]. Reduced levels of tricarboxylic acid (TCA) cycle intermediates such as succinate, together with decreased acetate, formate, and 3-hydroxybutyrate, are consistent with impaired mitochondrial oxidative metabolism and reduced β-oxidation [22,23]. Interestingly, although both ATP and ADP concentrations were markedly reduced, the ATP/ADP ratio was increased in the 1-week group, suggesting alterations in adenine nucleotide homeostasis rather than a simple depletion of high-energy phosphates. Concomitantly, increased glucose and lactate levels are consistent with a metabolic profile characterized by a greater reliance on glycolytic pathways [24]. Elevated lactate concentrations are known to reduce fatty acid oxidation [25,26].

The accumulation of amino acids, particularly branched-chain amino acids (isoleucine, valine, and leucine), as well as alanine and glutamine, suggests reduced mitochondrial catabolism and alterations in amino acid utilization. Increased inosine points to enhanced nucleotide degradation under conditions of energetic stress. Furthermore, elevated betaine levels may reflect changes in redox regulation. The absence of detectable NADP^+^ in the 1-week DEPs group could be consistent with this possibility. However, this should be interpreted cautiously, as NADP^+^ concentrations were below detection limits in this group and could not be accurately quantified. Collectively, these findings support metabolic reprogramming characterized by increased glycolytic reliance and alterations in cellular energy production and redox homeostasis.

Although the observed metabolite profile was consistent with a shift towards glycolytic metabolism, the underlying molecular mechanisms were not investigated in the present study. One potential contributor is HIF-1α, a master regulator of cellular adaptation to stress that promotes glycolytic metabolism and suppresses oxidative metabolic pathways [27,28]. The increases in myocardial glucose and lactate observed after one week of DEP exposure are compatible with a metabolic phenotype that could involve HIF-1α activation. However, assessment of HIF-1α expression or activity was out of the scope of this manuscript, and therefore its contribution remains speculative.

In plasma, metabolic changes were less pronounced than in myocardial tissue. Our discriminant analysis revealed a significant separation between groups, but the model validation yielded poor R^2^ and Q^2^ values and no individual metabolites showed statistically significant differences. This suggests that DEP exposure may induce subtle but coordinated changes across multiple plasma metabolites that become apparent only when considered collectively. Therefore, the plasma data should be interpreted as evidence of modest alterations in the overall metabolomic profile rather than as demonstrating specific changes in individual metabolites. This contrasts with previous studies reporting alterations in purine metabolites (IMP, inosine, hypoxanthine or adenosine) [7,10], amino acids such as arginine [9,10], and other metabolites including histidine and γ-linolenic acid [10]. The reasons for these discrepancies remain unknown but may relate to differences in exposure route, pollutant dose and duration of exposure.

^1^H-NMR spectroscopy is able to detect a restricted number of metabolites, with sensitivity influenced by magnetic field strength (9.4T in our case) as well as by intrinsic methodological limitations, since compounds present at very low concentrations may fall below the detection threshold [29]. Therefore, it cannot be excluded that additional metabolites, not detected in our analysis, may also be modulated by DEP exposure. Nevertheless, in comparison with other metabolomic approaches such as mass spectroscopy, NMR offers greater specificity and reproducibility and is less susceptible to variability introduced during sample handling, supporting the robustness of our data. An exception is targeted mass spectroscopy (LC-MS/MS) [30]. However, this methodology is typically restricted to the quantification of a limited panel of metabolites and remains unavailable in many laboratories. Additionally, pathway enrichment analyses were derived from a limited set of detected metabolites, and therefore should be considered complementary to the metabolite-level findings rather than direct evidence of pathway function. Further, pooled quality-control samples and technical replicates were not included in the present work. Although TSP was used as an internal concentration and chemical shift standard and spectral quality was verified before analysis, the absence of quality-control samples limited the formal assessment of analytical stability through this study.

Although OPLS-DA validation indicated good model performance, especially for myocardial samples, supervised multivariate approaches may be susceptible to overfitting when applied to relatively small datasets and should therefore be interpreted together with the univariate and pathway analyses. A further limitation of this study is that metabolic analysis was based on steady-state metabolite concentrations. Although changes in metabolite abundance and pathway enrichment analyses provide evidence of metabolic remodeling, they do not directly measure metabolic fluxes or enzymatic activities. Therefore, interpretations regarding altered glycolytic or mitochondrial metabolism should be made with caution, as the present metabolomic data do not directly measure metabolic fluxes or mitochondrial respiration. Nevertheless, the interpretation of altered mitochondrial metabolism is supported by recent observations in the same experimental model showing reduced citrate synthase activity, decreased mitochondrial DNA copy number, and increased mitochondrial ROS production following DEP exposure [19]. Additional metabolic flux and mitochondrial function studies will be required to establish a direct mechanistic link.

The absence of a time-matched saline control group for the 1-week exposure period does not allow us to exclude the possibility that a small proportion of the observed effects were influenced by time-dependent factors. However, in this regard, all animals originated from the same cohort, were age-matched at study initiation, and were maintained under identical housing and handling conditions. Moreover, the relatively short interval between the two time points and the attenuation of many metabolic alterations after 3 weeks of DEP exposure suggest that the observed metabolic changes are more likely related to DEP exposure than to experimental duration alone.

Although both male and female rats were included, the study was not designed or statistically powered to evaluate sex-specific effects. Consequently, potential sex-dependent differences in the myocardial metabolic response to DEP exposure cannot be excluded and warrant further investigation. Finally, another limitation of the study is that pulmonary function and systemic oxygenation were not assessed. Therefore, although no overt signs of respiratory dysfunction were observed during the experimental protocol, we cannot exclude the possibility that DEP-induced pulmonary alterations contributed to the cardiac metabolic changes observed through indirect mechanisms, including impaired gas exchange or tissue hypoxia.

Beyond identifying metabolic alterations, the present findings have potential pathophysiological relevance. The observed metabolic profile is consistent with reduced reliance on mitochondrial oxidative metabolism and increased dependence on glycolytic pathways, changes that may compromise myocardial energetic efficiency and contribute to the development of cardiac dysfunction. In the context of our previous observations of myocardial inflammation, oxidative stress, fibrosis and increased arrhythmia susceptibility following DEP exposure [2], these metabolic alterations may represent an additional mechanistic link between air pollution and adverse cardiovascular outcomes.

## 5. Conclusions

In conclusion, DEP exposure induces early, transient alterations in myocardial metabolic profile that are consistent with metabolic remodeling involving a shift from oxidative to glycolytic metabolism, together with disturbances in energy and redox homeostasis.

## Figures and Tables

**Table 1:** Quantification of myocardial metabolites (μmol/g fresh tissue) analyzed by ^1^H NMR spectroscopy in tissue extracts obtained from hearts from control rats and from animals exposed to DEPs for one or three weeks.

Metabolite	Control (n = 7)	DEPs (1 w) (n = 6)	DEPs (3 w) (n = 8)
3-Hydroxybutyrate	0.32 ± 0.04	0.18 ± 0.04	0.19 ± 0.03
Acetate	3.25 ± 0.53	0.24 ± 0.04 ***	3.41 ± 0.40 ###
ADP	1.46 ± 0.11	0.13 ± 0.01 ***	1.49 ± 0.12 ###
Alanine	1.60 ± 0.12	2.50 ± 0.12 ***	1.61 ± 0.11 ###
Aspartate	0.86 ± 0.07	1.04 ± 0.09	0.88 ± 0.03
ATP	1.44 ± 0.09	0.18 ± 0.01 ***	1.32 ± 0.10 ###
Betaine	0.18 ± 0.03	0.93 ± 0.05 ***	0.21 ± 0.02 ###
Creatine	12.11 ± 0.55	12.74 ± 0.67	12.26 ± 0.70
Formate	3.95 ± 0.49	0.49 ± 0.05 ***	3.67 ± 0.56 ###
Glucose	2.05 ± 0.31	4.74 ± 0.37 ***	1.80 ± 0.26 ###
Glutamate	5.42 ± 0.25	5.13 ± 0.22	5.17 ± 0.42
Glutamine	6.37 ± 0.27	7.84 ± 0.34 **	5.94 ± 0.38 ###
Inosine	0.39 ± 0.03	5.07 ± 0.21 **	0.36 ± 0.04 ##
Isoleucine	0.08 ± 0.01	0.18 ± 0.02 ***	0.06 ± 0.01 ###
Lactate	11.80 ± 0.59	22.99 ± 1.06 ***	12.04 ± 1.02 ###
Leucine	0.17 ± 0.02	0.23 ± 0.03 *	0.14 ± 0.01 ##
NADP^+^	0.63 ± 0.03	N.D.	0.58 ± 0.04
Phosphocreatine	0.48 ± 0.04	1.22 ± 0.45	0.49 ± 0.05
Propionate	0.16 ± 0.04	N.D.	0.18 ± 0.03
Succinate	0.98 ± 0.09	0.07 ± 0.02 **	0.81 ± 0.05 ##
Taurine	21.58 ± 0.08	18.62 ± 0.80	18.87 ± 1.55
Valine	0.13 ± 0.01	0.22 ± 0.03 **	0.11 ± 0.01 ###
ATP/ADP ratio	1.00 ± 0.04	1.35 ± 0.11 *	0.91 ± 0.07 ##

* (*p* < 0.05), ** (*p* < 0.01) and *** (*p* < 0.001) indicate a significant differences respect to hearts from control animals. ## (*p* < 0.01) and ### (*p* < 0.001) indicate significant differences vs. hearts treated with DEPs for one week after Benjamini–Hochberg FDR correction. N.D. indicates values below detection limit.

## Data Availability

The original contributions presented in this study are included in the article. Further inquiries can be directed to the corresponding authors.

## Abbreviations

The following abbreviations are used in this manuscript:

ANOVA	Analysis of variance
DEPs	Diesel exhaust particles
IP	Intraperitoneal
NMR	Nuclear magnetic resonance
PM	Particulate matter
OPLS-DA	Orthogonal partial least squares discriminant analysis
ROS	Reactive oxygen species
SEM	Standard error of the mean
TCA	Tricarboxylic acid cycle
TSP	3-(trimethylsilyl)propionic-2,2,3,3-d4 acid
